# Targeting Taxanes to Castration-Resistant Prostate Cancer Cells by Nanobubbles and Extracorporeal Shock Waves

**DOI:** 10.1371/journal.pone.0168553

**Published:** 2016-12-21

**Authors:** Francesca Marano, Letizia Rinella, Monica Argenziano, Roberta Cavalli, Francesca Sassi, Patrizia D’Amelio, Antonino Battaglia, Paolo Gontero, Ornella Bosco, Rossella Peluso, Nicoletta Fortunati, Roberto Frairia, Maria Graziella Catalano

**Affiliations:** 1 Department of Medical Sciences, University of Turin, Turin, Italy; 2 Department of Drug Science and Technology, University of Turin, Turin, Italy; 3 Department of Surgical Sciences, University of Turin, Turin, Italy; 4 Oncological Endocrinology, AO Città della Salute e della Scienza di Torino, Turin, Italy; Institute for Bioscience and Biotechnology Research, ITALY

## Abstract

To target taxanes to castration-resistant prostate cancer cells, glycol-chitosan nanobubbles loaded with paclitaxel and docetaxel were constructed. The loaded nanobubbles were then combined with Extracorporeal Shock Waves, acoustic waves widely used in urology and orthopedics, with no side effects. Nanobubbles, with an average diameter of 353.3 ± 15.5 nm, entered two different castration-resistant prostate cancer cells (PC3 and DU145) as demonstrated by flow cytometry and immunofluorescence. The shock waves applied increased the amount of intracellular nanobubbles. Loading nanobubbles with paclitaxel and docetaxel and combining them with shock waves generated the highest cytotoxic effects, resulting in a paclitaxel GI_50_ reduction of about 55% and in a docetaxel GI_50_ reduction of about 45% respectively. Combined treatment also affected cell migration. Paclitaxel-loaded nanobubbles and shock waves reduced cell migration by more than 85% with respect to paclitaxel alone; whereas docetaxel-loaded nanobubbles and shock waves reduced cell migration by more than 82% with respect to docetaxel alone. The present data suggest that nanobubbles can act as a stable taxane reservoir in castration-resistant prostate cancer cells and shock waves can further increase drug release from nanobubbles leading to higher cytotoxic and anti-migration effect.

## Introduction

Prostate cancer is the most common cancer in men [1]. Androgen-deprivation therapy is used to treat advanced or metastatic disease, however progression is common and leads to so called castration-resistant prostate cancer (CRPC) [2,3]. Patients with CRPC have a median survival rate of 12 months and to date there is no treatment that offers a survival advantage. Chemotherapy with taxanes (e.g. paclitaxel and docetaxel) is the main treatment for CRPC, but unfortunately this shows little effect on prolonging survival [4,5] and is complicated with several side effects [6].

Nanoscale microbubbles (nanobubbles, NBs) loaded with anticancer drugs, look like a promising delivery system [7] as they can carry loaded drugs through the blood to the tumor site, taking advantage of the enhanced permeability and retention effect (EPR) which is due to the defective vascular architecture of the tumor tissue [8,9]. Furthermore, under ultrasound exposure, NBs oscillate and collapse thus resulting in an increased intratumoral drug release [10,11]. Very recently, Fan et al. [12] demonstrated that ultrasound-triggered release of doxorubicin from NBs inhibited prostate cancer growth *in vitro* and *in vivo*.

Extracorporeal Shock Waves (ESWs), acoustic waves widely used in urology for lithotripsy [13], can be focused with high precision in depth and consequently able to determine the permeabilization of plasma membranes [14–16]. We recently reported elsewhere [17], that ESWs enhance cytotoxicity of doxorubicin-loaded glycol chitosan NBs in anaplastic thyroid cancer cells. Moreover, ESWs increase paclitaxel-induced apoptosis in breast cancer cell lines [14], in anaplastic thyroid cancer cells [18] and in a Mat B-III rat syngeneic model of breast cancer [19]. Lastly, they enhance the cytotoxic effect of doxorubicin and methotrexate in human osteosarcoma cell lines [20]. Hence, the combination of ESWs and taxane-loaded NBs may be an ideal drug delivery strategy for CRPC targeted therapy.

## Materials and Methods

### Preparation of the NBs

NBs were formulated by modifying the method previously reported [21]. Perfluoropentane was used as the inner core component and glycol chitosan for the shell. Firstly, an ethanolic solution containing palmitic acid and Epikuron 200^®^ (1% w/v) was added to the perfluoropentane to form a pre-emulsion. After the addition of ultrapure water, the system was homogenized using a high shear homogenizer (Ultraturrax^®^, IKA, Königswinter, Germany) in an ice-bath. Then to obtain polymeric NBs, an aqueous solution of the glycol chitosan polymer (2.7% w/v) was added drop-wise under mild magnetic stirring. To obtain the drug-loaded NBs, paclitaxel and docetaxel were dissolved in the perfluoropentane core using isopropanol and ethanol respectively as co-solvents, facilitating drug dissolution. Following this, the preparation protocol was carried out as previously reported [21]. The two NB formulations were purified by diaultrafitration, using a TCF2 system (Amicon) with a dialysis membrane cut off = 100 kDa, to remove the free drugs or other components in solutions.

Fluorescent NBs without any drug were obtained by adding green fluorescent 6-coumarin to the perfluoropentane core.

### Characterization of NB formulations

The average diameter and polydispersity index of NB formulations were determined by photon correlation spectroscopy (PCS); the zeta potential was determined by electrophoretic mobility using a 90 Plus instrument (Brookhaven, NY, USA). The analyses were performed at a scattering angle of 90° and a temperature of 25°C, using an NB suspension diluted with deionized distilled water. For zeta potential determination, samples of diluted NB formulations were placed in the electrophoretic cell where an electric field of approximately 15 V/cm was applied.

### Paclitaxel and docetaxel quantitative determination

A High Performance Liquid Chromatography (HPLC) analysis was purposely tuned modifying the method proposed by Kim et al. [22]. Paclitaxel and docetaxel quantitative determination was carried out by the HPLC system consisting of a Perkin Elmer pump (Perkin Elmer PUMP 250B, Waltham, MA) equipped with a spectrophotometer detector (Flexar UV/Vis LC spectrophotometer detector, Perkin Elmer, Waltham, MA). A reverse phase Agilent TC C18 column was used (150 cm ×4.6 mm, pore size 5 μm; Agilent Technologies, Santa Clara, CA, USA). The column was eluted with acetonitrile/water (60:40) at a flow rate of 1 ml/min. Paclitaxel and docetaxel were detected with a UV/vis detector at 227 and 230 nm respectively. The drug concentrations were calculated using an external standard method from standard calibration curves. Linear calibration curves were obtained over the concentration range of 0.5–25 μg/ml with a regression coefficient of 0.998. The sensitivity of this method reached a Limit of Quantitation (LOQ) value of 50 ng/ml for both the drugs.

### Encapsulation efficiency and loading capacity of taxane in NBs

The encapsulation efficiency of the two taxanes within the NB structure was determined using a centrifugal filter system. 150 μL of paclitaxel-loaded NB or docetaxel-loaded NB suspension were placed in an Amicon^®^ Ultra-0.5 centrifugal filter device and centrifuged at 15000 rpm for 30 minutes using a Beckman Coulter Allegra 64R Centrifuge. The ultrafiltrate obtained was quantified and after suitable dilution was analyzed by HPLC in order to obtain the concentration of free drug in NB suspensions. The encapsulation efficiency was calculated as the percentage of taxane incorporated in the NBs from the total amount of taxane initially used, according to the following equation: [(total amount of drug–amount of free drug)/total amount of drug] × 100.

The loading capacity was determined on freeze-dried NB samples. Briefly, a weighted amount of freeze-dried taxane-loaded NBs was diluted in 5 mL of ethanol. After sonication and centrifugation, the supernatant was analyzed by HPLC. The loading capacity of paclitaxel and docetaxel in the two NB formulations was calculated as [(total amount of drug–amount of free drug)/ weight of NBs] × 100.

### *In vitro* release studies

*In vitro* drug release experiments were conducted in a multi-compartment rotating cell, comprising a donor chamber & receiving chamber, kept separate by a cellulose membrane (Spectrapore, cut-off = 12000 Da). One ml of paclitaxel-loaded NB or docetaxel-loaded NB suspension was placed in the donor chamber. The receiving chamber contained 1 ml of phosphate buffer 0.05 M (pH 7.4) and 0.1% sodium dodecyl sulphate (SDS) was added to assure drug solubility. The receiving phase was withdrawn at regular intervals and completely replaced with the same amount of fresh buffer to maintain sink conditions. The concentration of paclitaxel and docetaxel in the withdrawn samples was detected by HPLC.

### Taxane loaded-NB stability over time

The physical stability of the two types of taxane-loaded NBs was evaluated by size and zeta potential determination of the two formulations over time and at different storage conditions (at either 4°C or 25°C). Their average diameters zeta potential values were assessed for a period of three months. Furthermore, the drug content of taxane-loaded nanobubbles was also investigated during this time (over the 3 months) by HPLC method, as described above.

### Cell lines and culture conditions

Two human metastatic prostate cancer cell lines, PC3 and DU145, were kindly provided by Stefania Pizzimenti (Dipartimento di Scienze Cliniche e Biologiche, Università di Torino). They are androgen-insensitive prostate cancer cells and used as representative of castration-resistant cancers. Cells were routinely maintained in 75 cm^2^ flasks at 37°C, in 5% CO_2_ and 95% humidity, with 100 IU/ml penicillin and 100 μg/ml streptomycin added in RPMI 1640 (GIBCO products Invitrogen Corp., Grand Island, NY, USA) supplemented with 10% fetal calf serum (FCS) (Euroclone, Wetherby, West York, UK)

### ESW treatment

The shock wave generator utilized for the *in vitro* experiments is a piezoelectric device (Piezoson 100, Richard Wolf, Knittlingen, Germany) designed for clinical use in orthopedics and traumatology. The experimental set-up has been reported elsewhere [14]. Aliquots of 1 ml of cell suspension adjusted to 1 x 10^6^ cells/ml were placed in 20 mm polypropylene tubes, completely filled with culture medium. Subsequently, cells were gently pelleted by centrifugation at 250 x *g* in order to minimize motion during shock wave treatment. Each cell-containing tube was placed in vertical alignment with the focal area and was adjusted so that the central point of the focal area corresponded to the center of the tube bottom. The shock wave unit was kept in contact with the cell containing tube by means of a water-filled cushion. Common ultrasound gel was used as a contact medium between cushion and tube. ESW treatment was as follows: energy flux density (EFD) = 0.59 mJ/mm^2^, 250 pulses (frequency = 4 shocks/s), peak positive pressure 64 MPa, peak negative pressure 12 MPa. After treatment, cell viability was evaluated in a hemocytometer chamber by a Trypan Blue dye exclusion assay.

### NB internalization

Cells were treated with 6-coumarin-labelled glycol chitosan NBs (15 x 10^4^ NBs/ml) alone or in combination with ESWs (0.59 mJ/mm^2^, 250 pulses); soon after they were seeded at 5 x 10^5^ cells/well in 6-well plates (Corning, New York, NY, USA). After 24 hours, green fluorescence in the cells was assessed by flow cytometer (EPICS XL, Coulter Corp., Hialeah, FL). Ten thousand events were acquired for each sample. In addition, cells treated as above were seeded at 3 x 10^3^ cells/well in 96-well plates. After 24 hours, cells were observed at a fluorescence microscope and photos were taken at x 200 magnification. ImageJ (version 1.48, NIH, Bethesda, Maryland) imaging software was used to quantify the amount of 6-coumarin-labelled glycol chitosan NBs in 10 fields for each condition.

### Cell viability assay

Cells were treated with: a) ESWs (0.59 mJ/mm^2^, 250 pulses); b) empty NBs (5–50 x 10^4^ NBs/ml); c) free paclitaxel (5–500 nM); d) free docetaxel (5–100 nM); e) paclitaxel-loaded NBs (5–500 nM); f) docetaxel-loaded NBs (5–100 nM); g) free paclitaxel plus ESWs; h) free docetaxel plus ESWs; i) paclitaxel-loaded NBs plus ESWs; l) docetaxel-loaded NBs plus ESWs. Cells in basal medium, RPMI 1640 plus 1% FCS, were used as controls. Cells were seeded at 3 x 10^3^ cells/well in 96-well plates (Corning, New York, NY, USA). After 24 hours, drug-containing medium was replaced with basal medium plus 1% FCS. Viable cells were determined at different times using the Cell Proliferation Reagent WST-1 (Roche Applied Science, Penzberg, Germany), following the manufacturer’s instructions. This is a colorimetric assay for the quantification of cell viability and proliferation based on the cleavage of the tetrazolium salt WST-1 by mitochondrial dehydrogenases. Briefly, 10 μl of WST-1 were added to each well. Then after 1 hour of incubation, absorbance at 450 nm was measured using a plate reader (Model 680 Microplate Reader, Bio-Rad, Hercules, CA, USA). Four replicate wells were used to determine each data point. Growth inhibition fifty (GI_50_), corresponding to the concentration of the compound that inhibits 50% cell growth, was calculated.

### Matrigel invasion assay

Cells were treated with: a) ESWs b) free paclitaxel (50 nM); c) free docetaxel (10 nM); d) paclitaxel-loaded NBs (50 nM); e) docetaxel-loaded NBs (10 nM); f) free paclitaxel plus ESWs; g) free docetaxel plus ESWs; h) paclitaxel-loaded NBs plus ESWs; i) docetaxel-loaded NBs plus ESWs. Cells in basal medium, RPMI 1640 plus 1% FCS were used as controls (Basal). Cells were seeded at 3 x 10^4^ cells/well in the upper chamber of Transwell Permeable Support (Corning, New York, NY, USA) in the presence of 1% FCS. The bottom chamber was filled with 0.6 ml RPMI 1640/ RPMI 1640 plus drugs with 10% FCS as a chemo attractant. After 24 hours, drug-containing medium was replaced with basal medium plus 1% FCS. After 48 hours from ESW treatment, cells on the upper side of the insert were removed with cotton swabs and cells on the lower surfaces of the membrane were fixed in methanol, stained with crystal violet and photographed. The number of cells that had migrated to the basal side of the membrane was quantified by counting 12 independent fields under the microscope.

### Statistical analysis

Data are expressed throughout the text as means ± SD, calculated from at least three different experiments. A comparison between groups was performed with an analysis of the variance (two-way ANOVA) and the threshold of significance was calculated with the Bonferroni test. Comparison between ESW-treated cells and no ESW-treated cells was performed with t-test. Statistical significance was set at p<0.05.

## Results

### Characterization of NB formulations

The average diameter, polydispersity index and Zeta-potential values of all NB formulations, before and after loading with paclitaxel or docetaxel, are reported in Table 1. The formulations showed average diameters of about 300 nm and a positive surface charge. The drug incorporation slightly affected the zeta potential values.

**Table 1:** Physico-chemical characteristics of empty, fluorescent and taxane-loaded NB formulations

Formulation	Average diameter ± SD (nm)	Polydispersity Index	Zeta Potential ± SD (mV)
Empty NBs	353.3 ± 15.5	0.214 ± 0.02	34.5 ± 3.8
Fluorescent NBs	345.6 ± 18.8	0.220 ± 0.02	34.1 ± 2.2
Paclitaxel-loaded NBs	325.8 ± 24.2	0.215 ± 0.01	27.6 ± 2.4
Docetaxel-loaded NBs	338.9 ± 22.4	0.228 ± 0.01	29.8 ± 3.5

Glycol chitosan NBs were able to load in the core the two taxanes to a large extent, showing an approximately 94% encapsulation efficiency and a loading capacity of approximately 5.8% for paclitaxel; docetaxel was incorporated in NB system with an encapsulation efficiency of about 86% and a loading capacity of about 5.2% respectively.

The release profiles of the paclitaxel-loaded NBs and docetaxel-loaded NBs were investigated *in vitro*. Prolonged *in vitro* release kinetics of the two taxanes from the drug-loaded NBs were demonstrated and no initial burst effect was observed for both the formulations. The results showed that about 15% of paclitaxel and 22% of docetaxel were released from the NBs within the first 8 hours. The sustained release of taxanes from the NB formulations at pH 7.4 proved encapsulation of the drug and the stability of the systems.

### NB stability over time

NBs formulations were evaluated for physical stability over time at different storage conditions, such as at 4°C or at 25°C. NBs formulations proved to be physically stable over time, as confirmed by long-term checking of the NB physico-chemical parameters. Indeed, the obtained values did not significantly change up to three months after preparation of the formulations. The storage conditions did not influence size (Table 2) and surface charge of the nanobubbles.

**Table 2:** Average diameter ± SD (nm) of the two taxane-loaded NB formulations over time at different storage conditions (either at 4°C or 25°C).

	Paclitaxel-loaded NBs		Docetaxel-loaded NBs	
Time (months)	Stored at 4°C	Stored at 25°C	Stored at 4°C	Stored at 25°C
0	325.8 ± 24.2	322.4 ± 18.7	338.9 ± 22.4	340.5 ± 17.6
1	333.2 ± 15.5	335.7 ± 20.3	342.3 ± 14.4	346.5 ± 19.6
2	340.7 ± 20.9	345.1 ± 16.6	348.4 ± 12.8	357.7 ± 18.2
3	352.4 ± 18.6	358.2 ± 22.1	355.6 ± 13.9	366.3 ± 22.5

The concentration of paclitaxel and docetaxel in the two NB formulations was maintained over time (Table 3).

**Table 3:** Drug content (%) of the two taxane-loaded NB formulations over time at different storage conditions (either at 4°C or 25°C).

	Paclitaxel-loaded NBs		Docetaxel-loaded NBs	
Time (months)	Stored at 4°C	Stored at 25°C	Stored at 4°C	Stored at 25°C
0	100	100	100	100
1	98.55	98.42	97.63	97.70
2	96.90	96.84	95.15	94.89
3	95.11	94.77	93.90	93.72

### ESWs and empty NBs are safe

**Fig 1:**
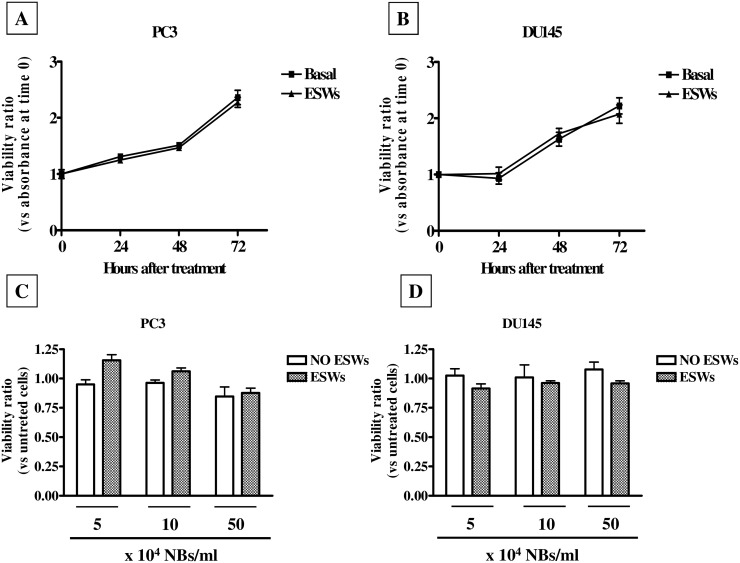
Effect of ESWs and empty NBs on cell viability. Growth curve of PC3 (A) and DU145 (B) cells after treatment with ESWs (250 pulses at 0.59 mJ/mm^2^) expressed as viability ratio vs time 0. Viability of PC3 (C) and DU145 (D) cells treated for 72 hours with empty NBs (5–50 x 10^4^ NBs/ml), either in the presence or absence of ESWs. Viability is expressed as ratio between absorbance of treated cells and absorbance of untreated cells (abs = 0.367 ± 0.015 for PC3; abs = 0.221 ± 0.009 for DU145).

In a preliminary series of experiments cells were treated with ESWs, using 250 pulses at EFD = 0.59 mJ/mm^2^, in order to exclude any effects of shock waves alone on cell viability and growth. The used energy was within the range of clinical use; in fact, in the extracorporeal shockwave therapy ESWs are applied ranging from 0.01 to 0.6 mJ/mm^2^ [23]. Soon after treatment, cell viability of both PC3 and DU145 cell lines was higher than 85% (data not shown). Moreover, the same treatment did not affect cell growth of both cell lines up to 72 hours (Fig 1A and 1B). Therefore, this treatment schedule was chosen for further experiments.

As the shell influences NB characteristics and behavior, we tested whether empty NBs affected cell viability of both prostate cell lines. Empty NBs had no significant effect on cell viability of both cell lines even when used in combination with ESWs (Fig 1C and 1D), and this allowed us to exclude any non-specific effect of the combined treatment on cell viability.

### ESW effect on the entry of NBs inside the cells

**Fig 2:**
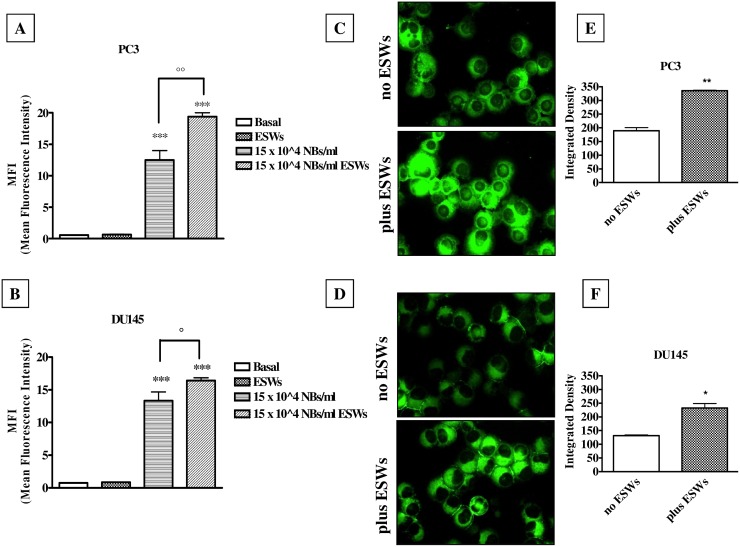
NB entrance. Cytofluorimetric analysis of PC3 (A) and DU145 (B) cells treated for 24 hours with 6-coumarin-labelled glycol chitosan NBs (15 x 10^4^ NBs/ml), either in the absence and in the presence of ESWs, expressed as Mean Fluorescence Intensity (MFI). Significance *vs* untreated cells (Basal): p<0.001 (***); significance vs ESWs: p<0.05 (°); p<0.01 (°°). Photos by fluorescence microscope of PC3 (C) and DU145 (D) cells treated with 6-coumarin-labelled glycol chitosan NBs at 15 x 10^4^ NBs/ml, either in the absence and in the presence of ESWs. Pictures were taken at 200 x final magnification (scale bar: 100 μm). The images are representative of three independent experiments; for each experiment, 10 fields were quantified. Image-based quantification of 6-coumarin-labelled glycol chitosan NBs in PC3 (panel E) and DU145 (panel F) cells. Significance *vs* no ESWs: p<0.05 (*); p<0.01 (**).

In both cell lines, NBs entered the cells (p<0.001) and ESW treatment further increased the amount of intracellular NBs (in PC3, p<0.01; in DU145, p<0.05), as shown in Fig 2A and 2B. Moreover, NBs were visible inside the cells (Fig 2C and 2D), and the fluorescence intensity was significantly increased by ESW treatment (in PC3, p<0.01, Fig 2E; in DU145, p<0.05, Fig 2F).

### ESW effect on paclitaxel cytotoxicity

**Fig 3:**
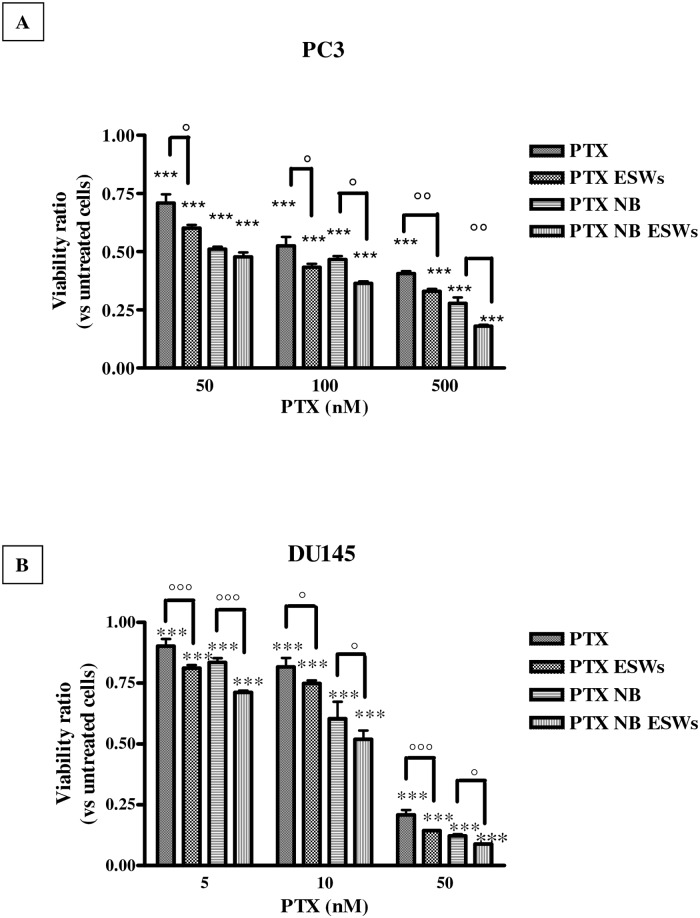
Paclitaxel cytotoxicity. Cytotoxic effects of paclitaxel (PTX), paclitaxel + ESWs (PTX ESWs), paclitaxel-NBs (PTX NB), and paclitaxel-loaded NBs + ESWs (PTX NB ESWs), in PC3 (panel A) and in DU145 (panel B) cells. Viability is expressed as ratio vs untreated cells after a 72 hour treatment (abs = 0.804 ± 0.065 for PC3; abs = 0.548 ± 0.039 for DU145). Significance *vs* untreated cells: p<0.001 (***); significance *vs* no ESWs: p<0.05 (°), p<0.01 (°°); p<0.001 (°°°).

The cytotoxic effects of paclitaxel in PC3 and DU145 cells are reported in Fig 3, panels A and B, and Table 4. After 72 hours, free paclitaxel exerted some cytotoxic effect on both cell lines with a GI_50_ of 349.5 ± 4.8 nM for PC3 cells, and 163.3 ± 5.0 nM for DU145 cells, respectively (Table 1). ESW treatment increased the cytotoxic effects of the free drug; in fact GI_50_ decreased to 269.9 ± 16.2 nM for PC3 (p<0.01), and to 114.6 ± 5.8 nM for DU145 (p<0.05). Loading the drug into NBs, resulted in a significantly higher cytotoxic effect, being GI_50_ = 242.5 ± 8.5 nM for PC3 (p<0.01) and 109.0 ± 11.8 nM for DU145 (p<0.05), respectively. However, it was the combination of paclitaxel-loaded NBs and ESWs that showed the best effect; in fact, in PC3 GI_50_ further decreased to 180.1 ± 9.2 nM (significance *vs* free paclitaxel, p<0.001; significance *vs* paclitaxel-loaded NBs, p<0.05) and in DU145 GI_50_ decreased to 63.4 ± 8.5 nM (significance *vs* free paclitaxel, p<0.01; significance *vs* paclitaxel-loaded NBs, p<0.05).

**Table 4:** GI_50_ in prostate cancer cells for paclitaxel and paclitaxel-loaded NBs in the presence or absence of ESWs.

	GI_50_ (nM)			
	PC-3		DU145	
*Treatments*	*NO ESWs*	*ESWs*	*NO ESWs*	*ESWs*
**Paclitaxel**	349.5 ± 4.8	269.9 ± 16.2 (**) (°°)	163.3 ± 5.0	114.6 ± 5.8 (*) (°)
**Paclitaxel-loaded glycol chitosan NBs**	242.5 ± 8.5 (**)	180.1 ± 9.2 (***) (°)	109.0 ± 11.8 (*)	63.4± 8.5 (**) (°)

Significance vs Paclitaxel:

*, p<0.05;

**, p<0.01;

***, p<0.001

Significance vs NO ESWs:

°, p<0.05;

°°, p<0.01

### ESW effect on docetaxel cytotoxicity

**Fig 4:**
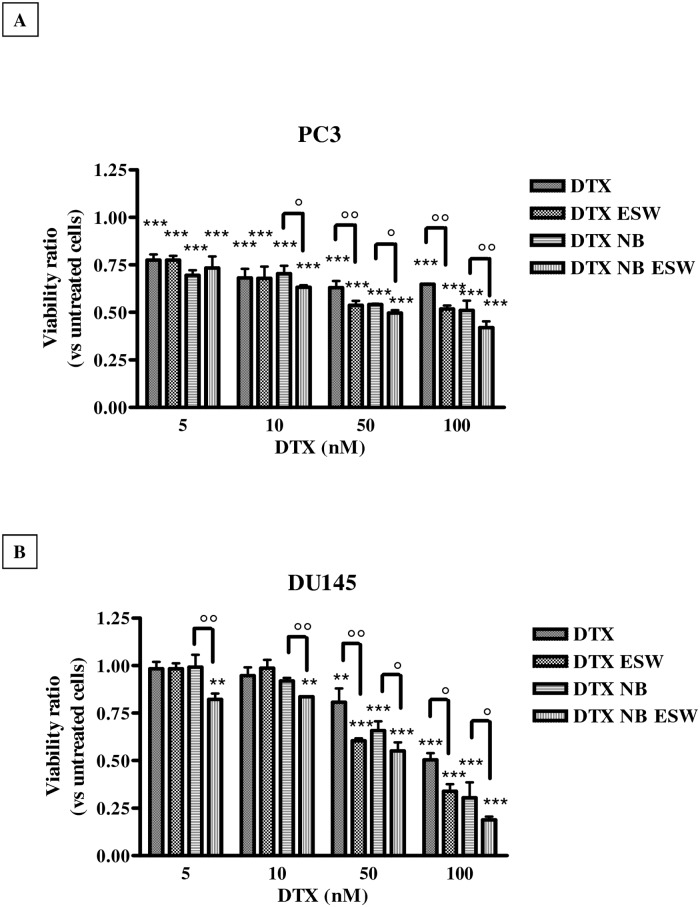
Docetaxel cytotoxicity. Cytotoxic effects of docetaxel (DTX), docetaxel + ESWs (DTX ESWs), docetaxel-NBs (DTX NB), and docetaxel-loaded NBs + ESWs (DTX NB ESWs), in PC3 (panel A) and DU145 (panel B) cells. Viability is expressed as ratio vs untreated cells at 48 hours after treatments (abs = 0.399 ± 0.025 for PC3; abs = 0.332 ± 0.039 for DU145). Significance vs untreated cells: p<0.01 (**), p<0.001 (***); significance vs no ESWs: p<0.05 (°), p<0.01 (°°).

The cytotoxic effects of docetaxel in PC3 and DU145 cells are reported in Fig 4, panels A and B, and Table 5. GI_50_ of free docetaxel was 126.6 ± 1.2 nM in PC3 cells, and 91.4 ± 0.6 nM for DU145 cells, respectively (Table 5). The cytotoxic effects of the free drug were increased by ESW treatment, in fact GI_50_ decreased to 89.9 ± 7.8 nM for PC3 (p<0.01), and to 73.5 ± 4.1 nM for DU145 (p<0.05). Loading the drug into NBs, resulted in a significantly higher cytotoxic effect, being GI_50_ = 96.7 ± 1.6 nM for PC3 (p<0.01) and 74.9 ± 1.7 nM for DU145 (p<0.05), respectively. Again, it was the combination of docetaxel-loaded NBs and ESWs that showed the best cytotoxic effect; in fact in PC3, GI_50_ further decreased to 66.2 ± 6.8 nM (significance *vs* free docetaxel, p<0.001; significance *vs* docetaxel-loaded NBs, p<0.05) and in DU145, GI_50_ decreased to 55.2 ± 0.18 nM (significance *vs* free docetaxel, p<0.01; significance *vs* docetaxel-loaded NBs, p<0.05).

**Table 5:** GI_50_ in prostate cancer cells for docetaxel and docetaxel-loaded NBs in the presence or absence of ESWs.

	GI_50_ (nM)			
	PC3		DU145	
*Treatments*	*NO ESWs*	*ESWs*	*NO ESWs*	*ESWs*
**Docetaxel**	126.6 ± 1.2	89.9 ± 7.8 (**) (°°)	91.4 ± 0.6	73.5 ± 4.1 (*) (°)
**Docetaxel-loaded glycol chitosan NBs**	96.7 ± 1.6 (**)	66.2 ± 6.8 (***) (°)	74.9 ± 1.7 (*)	55.2 ± 0.18 (**) (°)

Significance vs Docetaxel:

*, p<0.05;

**, p<0.01;

***, p<0.001

Significance vs NO ESWs:

°, p<0.05;

°°, p<0.01

### ESW effect on cell migration

**Fig 5:**
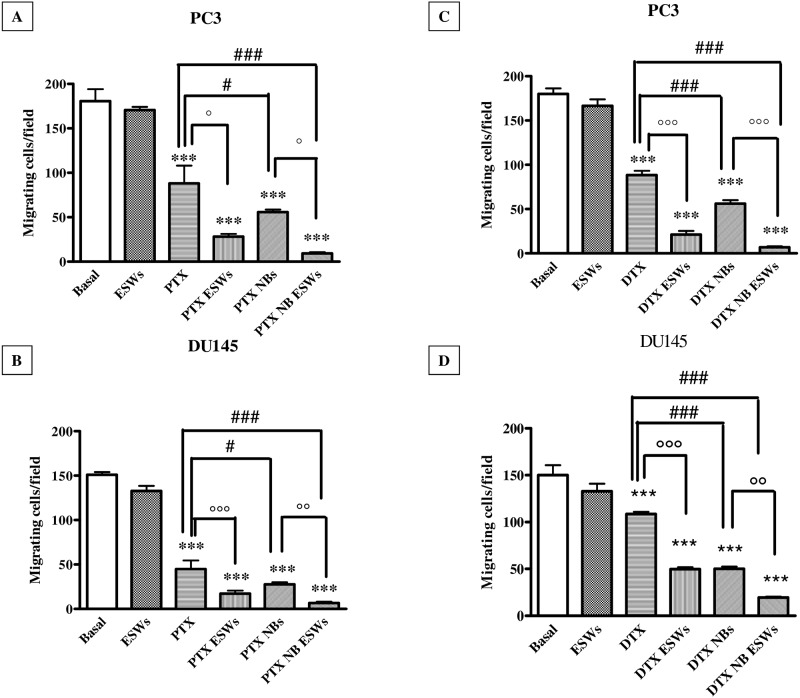
Taxane effects on cell migration. Matrigel invasion assay in PC3 (panels A and C) and DU145 (panel B and D) cells. Number of cells/field invading onto the lower surfaces of the filter was counted. Significance vs untreated cells (Basal): p<0.001 (***); significance vs no ESWs: p<0.05 (°), p<0.01 (°°), p<0.001 (°°°). Significance vs free drug: p<0.05 (#), p<0.001 (###).

As taxanes affect cell migration by acting on microtubule stabilization [24] the effect of different treatments on the invasive potential of prostate cancer cells was assessed by a transwell invasion chamber. As shown in Fig 5, the treatment with either free paclitaxel or docetaxel reduced the number of migrating PC3 cells of 50.9% (p<0.001) with respect to untreated cells (panels A and C). In DU145 cell, paclitaxel (panel B) reduced the number of migrating cells of 70.4% (p<0.001), but docetaxel (panel D) determined a reduction of only 27.6% (p<0.001). Treatment with either ESWs or drug-loaded NBs further increased the inhibition effect of paclitaxel and docetaxel in both cell lines. Indeed, adding ESWs or loading paclitaxel into NBs decreased the number of migrating PC3 cells of 68.2% (p<0.05) and 36.7% (p<0.05) respectively, compared to free paclitaxel (panel A). In DU145 cells (panel B), ESWs decreased migration of 61.9% (p<0.001), and paclitaxel-loaded NBs determined a reduction of 38.6% (p<0.05), with respect to paclitaxel alone. As far as docetaxel is concerned, the number of PC3 migrating cells was decreased of 75.9% (p<0.001) and 36.6% (p<0.001) by ESWs and docetaxel-loaded NBs respectively, compared to the effect of the drug alone (panel C). In DU145 (panel D) ESWs determined a 54.2% decrease in migration (p<0.001) and loading docetaxel in NBs induced a 53.4% decrease (p<0.001) of migrating cells with respect to cells treated with docetaxel alone. But, again, it was the combination of drug-loaded NBs and ESWs that elicited the best effects in terms of inhibition of cell migration. For paclitaxel, PC3 migrating cells were decreased of 89.4% (panel A, p<0.001) and DU145 migrating cells were decreased of 85.1% (panel B, p<0.001) with respect to cells treated with paclitaxel alone. For docetaxel, PC3 migrating cells were decreased of 92.3% (panel C, p<0.001) and DU145 migrating cells were decreased of 82.2% (panel D, p<0.001) with respect to cells treated with docetaxel alone.

## Discussion

The present study for the first time demonstrates that the combined treatment with both paclitaxel- or docetaxel-loaded NBs and ESWs enhances cytotoxicity of taxanes in human castration-resistant prostate cancer cells. Paclitaxel and, more recently, docetaxel are the most used anticancer drugs for prostate cancer treatment [25]. However, the onset of resistance and the presence of side effects commonly result in modest survival gains. Therefore, new drug formulations with tumor-selective delivery may readdress these limitations. Nanotechnology is a promising tool to enhance antitumor efficacy of cytotoxic drugs. Targeting anticancer agents to tumor tissues, in fact, has demonstrated increased tumor localization, resulting in significant enhancements of therapeutic effectiveness [26]. Taxanes have been encapsulated into different kinds of nanoparticles [27]. The most widely used class of biocompatible and biodegradable polymers, approved by Food and Drug Administration, is that of aliphatic polyesters, including poly(lactic acid) (PLA), poly(glycolic acid) (PLGA), and their copolymers [28,29]. Moreover, nanoparticles decorated with the shell surface RNA A10-Aptamer (Apt), able to bind prostate-specific membrane antigen (PSMA), have been proposed [30]. Present data describe a new combination treatment modality that combines NBs and ESWs to target and release taxanes into prostate cancer cells, endorsing our previous observation on the combination of doxorubicin-loaded NBs and ESWs in anaplastic thyroid cancer cells [17]. In fact, combination of ESWs and NBs enhanced paclitaxel cytotoxicity, decreasing the drug GI_50_ of 52% in PC3 cells and of 61% in DU145 cells, respectively. A similar effect was obtained also for docetaxel activity; in fact, the drug GI_50_ decreased of 48% in PC3 cells and of 40% in DU145 cells. Moreover, this suggested new combination therapy was effective not only on cell viability but also on cell migration, where a huge reduction of the invasive cell potential was observed. In fact, combined treatment with paclitaxel-loaded NBs and ESWs reduced cell migration of more than 85% with respect to treatment with paclitaxel alone; whereas docetaxel-loaded NBs and ESWs reduced cell migration of more than 82% with respect to treatment with docetaxel alone. The enhancement of cytotoxicity and migration inhibition may be the consequence of cavitation which occurs when acoustic waves propagate into fluids [31]. In fact, cavitation determines both transient permeabilization of plasma membranes, thus allowing drug penetration into the cells, as well as the perturbation of the NBs, resulting in the intracellular drug release. Indeed, we here demonstrated that NB entrance was significantly increased by ESW treatment. Accordingly, it has already been reported that ESWs increase cell permeability [15,16,18], affecting drug efficacy in breast cancer cells [14], in anaplastic thyroid cancer cells [18], and in an *in vivo* breast cancer model [19]. Therefore, NBs can act as stable taxane reservoir in the castration-resistant prostate cancer cells and ESWs further increase drug release from NBs, leading to higher cytotoxic and anti-migration effects. This study suggests that, beyond ultrasounds, other physical forces can be used to trigger taxane release form NBs. Accordingly, very recently, Zhong et al. demonstrated, very recently, that upon pulsed laser irradiation, paclitaxel loaded nanoparticles induced apoptosis in prostate cancer cells and inhibited tumor growth *in vivo* [32].

In conclusion, this first *in vitro* study on the use of ESWs and taxane-loaded NBs, advises that this combined treatment may be a promising drug delivery tool for targeted treatment of castration-resistant prostate cancer; further characterization in preclinical *in vivo* models will define the possibility of its use in clinical practice.
